# Current News

**Published:** 1894-06

**Authors:** 


### Current News.

### PENNSYLVANIA STATE DENTAL EXAMINING BOARD.

THE Pennsylvania State Dental Examining Board will meet for the transaction of business at Cresson, Pa., commencing on Tuesday, July 10, 1894.

W. E. MAGILL, Erie, Pa.,
*President.*
J. C. GREEN, West Chester, Pa.,
*Secretary.*

### CHICAGO DENTAL SOCIETY.

AT the annual meeting of the Chicago Dental Society, held in Kindergarten Hall, April 3, 1894, the following officers were duly elected for the ensuing year:

J. H. Wolley, President; C. E. Bentley, First Vice-President; D. M. Gallic, Second Vice-President; A. H. Peck, Recording Secretary; H. A. Costner, Corresponding Secretary; E. D. Swain, Treasurer; J. J. Whaley, Librarian; J. N. Crouse, Director.

HENRY A. COSTNER,
*Corresponding Secretary.*

### MASSACHUSETTS DENTAL SOCIETY.

THE Twenty-ninth Annual Meeting of the Massachusetts Dental Society will be held June 7 and 8, 1894, in Massachusetts Institute of Technology Building, Boston. A very interesting meeting is promised. Dr. J. N. Crouse, of Chicago, is to be a guest of the Society, and will deliver an address. There is to be a banquet Thursday evening, at which Governor Greenhalge, of Massachusetts, and President Eliot, of Harvard, are expected to be present. A cordial invitation to be present is extended to members of the profession.

EDGAR O. KINSMAN,

*Secretary.*

CAMBRIDGE, MASS.

### FIRST DISTRICT DENTAL SOCIETY, STATE OF NEW YORK.

At the annual meeting of the First District Dental Society of the State of New York, held April 10, 1894, the following officers were elected for the ensuing year:

Victor Hugo Jackson, President; James W. Taylor, Vice-President; Benjamin C. Nash, Secretary; John H. Meyer, Treasurer; and J. Bond Littig, Librarian. Delegates to the Dental Society of the State of New York, George Evans and George H. Rich, each for four years, and M. L. Rhein for one year.

B. C. Nash,
*Secretary.*

---

### CONNECTICUT VALLEY DENTAL SOCIETY.

The Thirty-first Annual Meeting of the Connecticut Valley Dental Society will be held at Holyoke, Mass., June 20, 21, and 22. A cordial invitation is extended to members of dental societies.

Geo. A. Maxfield, D.D.S.

